# Sequential tyrosine kinase inhibitor therapy for EGFR L747P-mutated lung adenocarcinoma in a renal transplant recipient: a 24-month case report

**DOI:** 10.3389/fmed.2025.1651584

**Published:** 2025-10-08

**Authors:** Wenxiu Xie, Feng Chen, Lei Zhang, Baoquan Lin, Jia Ye, Zongyang Yu, Lei Gu, Wei Liu

**Affiliations:** ^1^Department of Respiratory and Critical Care Medicine, Fuzong Teaching Hospital of Fujian University of Traditional Chinese Medicine (900th Hospital), Fuzhou, China; ^2^Liancheng County Hospital, Longyan, Fujian, China; ^3^Department of Respiratory and Critical Care Medicine, 900th Hospital of PLA Joint Logistic Support Force, Fuzong Clinical Medical College of Fujian Medical University, Fuzhou, China; ^4^Department of Respiratory and Critical Care Medicine, School of Medicine, 900th Hospital of PLA Joint Logistic Support Force, Dongfang Hospital of Xiamen University, Xiamen University, Fuzhou, China; ^5^Department of Cardiothoracic Surgery, 900th Hospital of PLA Joint Logistic Support Force, Fuzong Clinical Medical College of Fujian Medical University, Fuzhou, China; ^6^Department of Pulmonary and Critical Care Medicine, The First Affiliated Hospital of Soochow University, Suzhou, China; ^7^Institute of Respiratory Diseases, Soochow University, Suzhou, China; ^8^Suzhou Key Laboratory for Respiratory Diseases, Suzhou, China

**Keywords:** kidney transplant recipients, lung adenocarcinoma, EGFR L747P mutation, tyrosine kinase inhibitors, sequential therapy, case report

## Abstract

**Background:**

Kidney transplant recipients (KTRs) are at increased risk of malignancies, including lung adenocarcinoma (LUAD). The therapeutic efficacy of tyrosine kinase inhibitors (TKIs) against the rare EGFR L747P mutation remains controversial.

**Case presentation:**

We report a 60-years-old female renal transplant recipient who developed advanced lung adenocarcinoma harboring the EGFR L747P mutation.

**Management and outcomes:**

The patient was treated sequentially with three generations of EGFR TKIs–gefitinib, osimertinib, and dacomitinib–while continuing immunosuppressive therapy for graft function. Gefitinib achieved a progression-free survival (PFS) of 9 months, osimertinib 4.5 months, and dacomitinib 2.5 months, resulting in a total overall survival (OS) of 24 months. Treatment was generally well tolerated, with only mild adverse events and manageable renal function changes.

**Conclusion:**

To our knowledge, this is the first reported case of an EGFR L747P-mutated lung adenocarcinoma in a renal transplant recipient benefiting from sequential multi-TKI therapy. This case underscores the importance of vigilant cancer surveillance in transplant recipients and suggests that individualized TKI sequencing may offer clinical benefit, although further evidence is required.

## Introduction

Kidney transplant recipients have a significantly increased risk of developing malignancies compared to the general population, partly due to long-term immunosuppression (1, 2). Among these, lung cancer is one of the most common and accounts for 3%–7% of *de novo* cancers after kidney transplantation (1, 3, 4).

In China, lung adenocarcinoma is the predominant histological subtype, with up to 50% of patients harboring EGFR mutations such as exon 19 deletion or exon 21 L858R substitutions (5). While EGFR tyrosine kinase inhibitors are the standard first-line therapy for EGFR-mutant LUAD, the clinical response to rare mutations remains uncertain. In particular, the exon 19 L747P mutation has shown inconsistent sensitivity to TKIs across different generations, with case reports describing variable efficacy of first-, second-, and third-generation agents (6–13).

Here, we present a rare case of a non-smoking female renal transplantation recipient with advanced LUAD harboring the EGFR L747 P mutation. She was sequentially treated with three generations of TKIs and achieved an overall survival of 24 months. This case highlights the therapeutic challenges posed by this rare mutation and provides clinical evidence for individualized treatment strategies in the transplant setting.

## Case report

**FIGURE 1:**
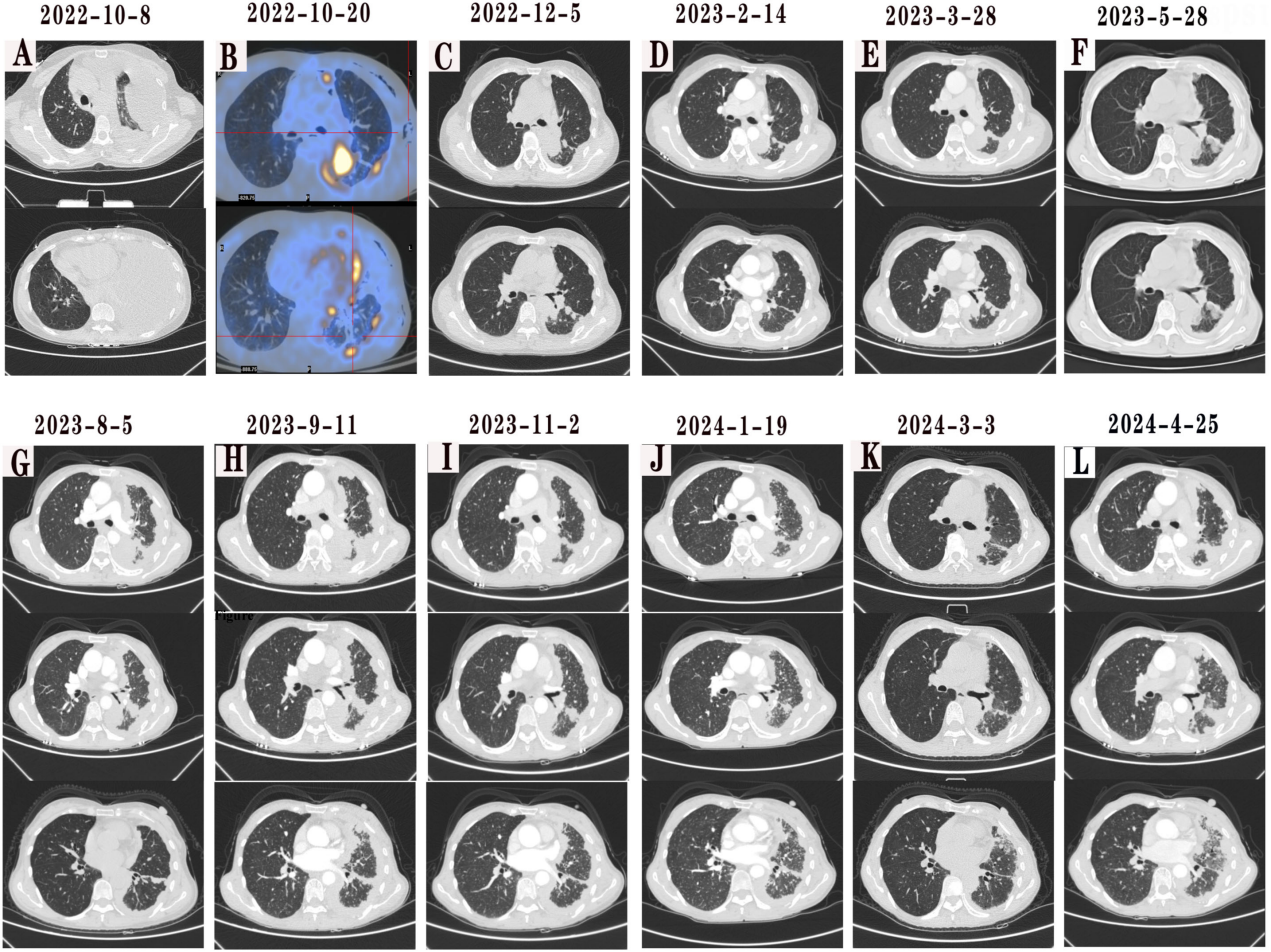
**(A)** On admission (October 8, 2022), CT scan showed left pneumonia with massive effusion of the left lung and left lung atelectasis. **(B)** On October 20, 2022, 18F-FDG PET-CT scan were performed after thoracoscopic surgery and showed soft tissue density mass near the left upper lung portal. **(C–F)** After starting first-line treatment with gefitinib, chest CT scans on February 14, 2023, March 28, 2023, and May 28, 2023, showed that tumor size was similar to that on December 5, 2022. **(G)** On August 5, 2023, chest CT scan showed left hilar carcinoma with left pleural tumor metastasis and left pleural effusion progression. **(H–J)** From September 2023, the patient was switched to osimertinib as second-line drug, on November 2, 2023 chest CT scan indicated slow progression of the chest lesion, while January 19, 2024 chest CT scan showed that the metastatic pulmonary nodules in the right lung were found to increase significantly. **(K)** On February 2, 2024, we replaced the patient with dacomitinib for treatment, on March 3, 2024 chest CT scan showed that the metastatic tumors in the left pleura and the metastatic tumors in the right lung were found to shrink significantly. **(L)** However, on April 25, 2024, a follow-up chest enhanced CT scan showed that the lesion had progressed again.

**FIGURE 2:**
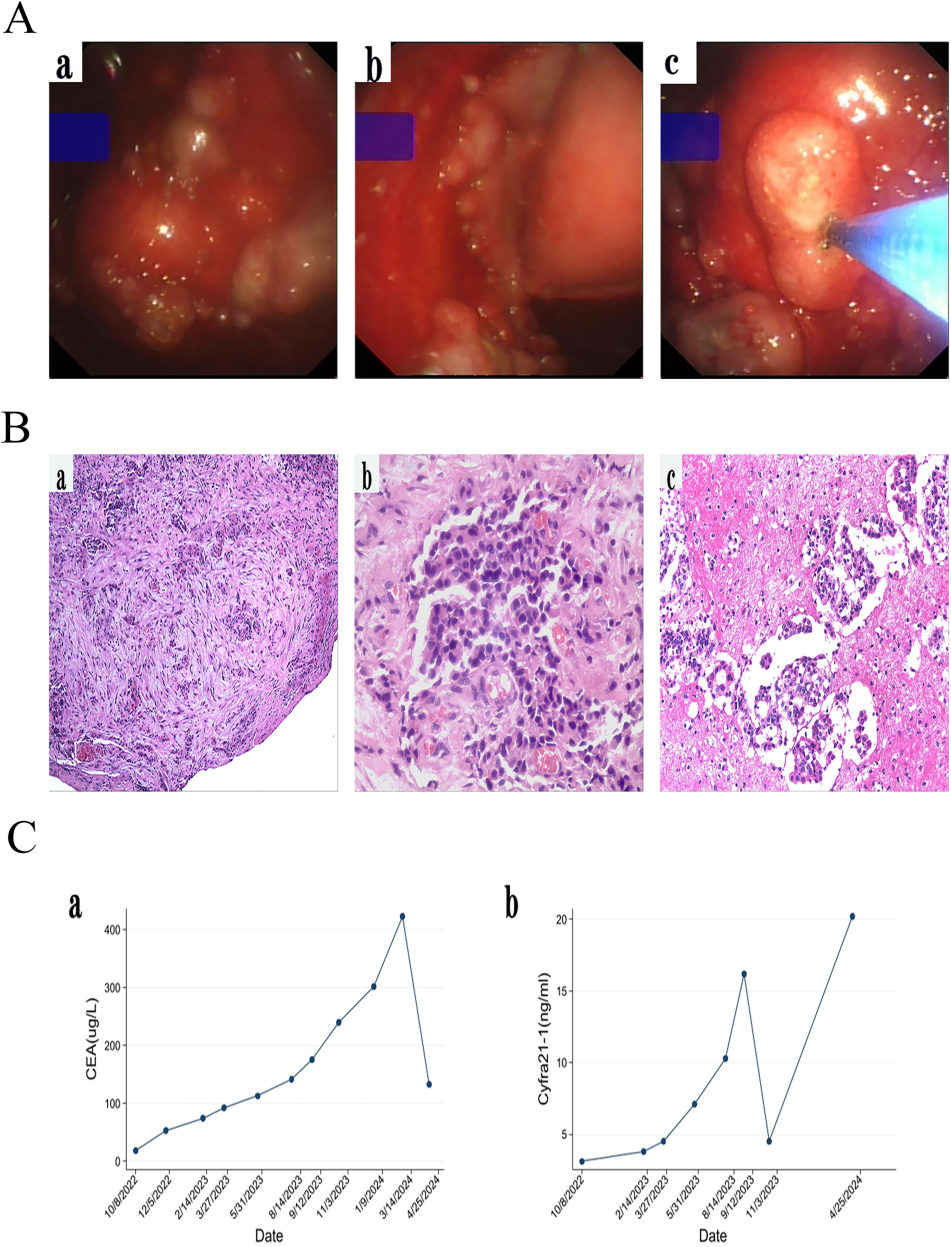
**(A)** Medical thoracoscopic pleural biopsy showed the visceral pleura and parietal pleura were full of new organisms of different sizes, and the new organisms were biopsied. **(B)** Histological examination (HE) revealed a small number of cellularly heterogeneous cells in pleural fibrous connective tissue (a,b) and in pleural effusion (c). **(C)** CEA and Cyfra21-1 gradually increased during follow-up of gefitinib. However, CEA and Cyfra21-1 has decreased since we replaced the patient with the second and third generation TKI for treatment.

A 60-years-old female recipient of a living relative kidney transplant for end-stage renal disease in early July 2016 was admitted to our hospital on October 8, 2022 presenting with cough, sputum production, chest tightness, and shortness of breath persisting for over 2 months. Thoracic color Doppler ultrasound revealed massive pleural effusion. The patient underwent regular follow-up in the kidney transplant clinic of our hospital, and her renal function remained normal at the time of this admission. The immunosuppression regimen consisted of mycophenolate mofetil (250 mg, twice daily), tacrolimus (0.5 mg, twice daily), and prednisolone (5 mg, once daily). The patient denied a history of smoking and any family history of lung cancer or other malignancies. During admission, routine laboratory tests were conducted on blood, sputum, pleural fluid, urine, and stool samples. Additionally, chest computerized tomography (CT) scan (Figure 1A), medical thoracoscopy with pleural biopsy (Figure 2A) were performed. It was determined that she had type 1 respiratory failure [Arterial blood gas was analyzed (FiO2 = 0.21), and the results were as follows: pH of 7.43, partial pressure of oxygen (PO2) of 69 mmHg, partial pressure of carbon dioxide (pCO2) of 31 mmHg, standard bicarbonate (HCO3-std) of 22.7 mmol/L, oxygen saturation (SaO2) of 97%, and oxygenation index of 238] with non-infectious exudative pleural effusion. Based on the laboratory results from blood and pleural fluid (Table 1) analysis along with histological examination findings of pleural fluid cells and tissue showing cancer cells (Figure 2B), confirmed as LUAD through immunohistochemistry staining (Table 2). Subsequently, to comprehensively evaluate the lung cancer staging, the patient underwent brain magnetic resonance imaging (MRI) and 18F-FDG positron emission tomography-CT (PET-CT) scan (Figure 1B). Chest CT scan findings revealed a soft tissue density mass near the left upper lung hilum with high uptake of 18F-FDG (SUV max 9.5), accompanied by multiple lymph node metastases in the left hilus of the lung, mediastinum, and retroperitoneal para-aorta; as well as left pleural and pericardial metastases resulting in left-sided pleural effusion and pericardial effusion. No metastasis was observed in the central nervous system, liver, spleen transplanted kidney, and bone. Furthermore, mutation status of 10 driver genes in matched tumor samples was detected by a next generation sequencing (NGS) panel (Amoy Diagnostics), which can detect hotspot mutations/fusions in genes of EGFR, KRAS, BRAF, NRAS, HER2, PIK3CA, ALK, ROS1, RET, and MET in one single test. Analysis of the pleural tissues identified an EGFR exon19 c2239_2240 delinsCCp. (L747P) NM_005228.5 mutation. Considering all these results, the patient was diagnosed with stage IV LUAD (T2aN2M1c) harboring a rare EGFR L747P mutation.

**TABLE 1:** Results of the pleural fluid test.

Surveillance project	October 9,2022	October 12,2022	Normal reference values
Color	yellow	yellowish	yellowish
Transparency	Turbid	Slightly turbid	Clear
Red blood cell (RBC)	1.9×10^9^/L	4.1×10^9^/L	–
White blood cell (WBC)	0.625×10^9^/L	0.455×10^9^/L	–
Multinucleate cells (MNC)	5%	11%	–
Monocytes (Mo)	95%	89%	–
Rivalta test	++	++	negative
Glucose	5.68 mmol/L	8.04 mmol/L	3.63∼4.78 mmol/L
Cl	111.8 mmol/L	107.0 mmol/L	95∼106 mmol/L
Lactate dehydrogenase (LDH)	175.9 U/L	231.5 U/L	40∼130 U/L
Adenosine deaminase (ADA)	6.8 U/L	7.6 U/L	0∼25 U/L
Total protein (TP)	39.2 g/L	40.0 g/L	5∼30 g/L
Carcinoembryonic antigen	73.01 ng/ml	74.08 ng/ml	Non-smokers <5 ng/ml | Smokers <5.5 ng/ml | Smokers and over 40 <65g/m
Sugar antigen CA12-5	477.8 U/ml	419.50 U/ml	<35 U/m
Sugar antigen CA19-9	/	8.24 U/ml	<39 U/ml
Sugar antigen CA 15-3	/	62.24 U/ml	<25 U/ml
Ferritin	/	557.3 ng/ml	20-400 ng/ml for males | 13–150 ng/ml for females
Acid-fast-bacillus	negative	negative	negative
Bacteria	negative	negative	negative
Fungus	negative	negative	negative

+: positive, –: indicates no data, /: the test has not been carried out.

**TABLE 2:** Immunohistochemical results of pleural fluid cells and pleural biopsy specimens.

Specimen (date)	October 14, 2022	October 18, 2022	October 25, 2022
**Antibody type**	**Hydrothorax**	**Hydrothorax**	**Pleura**
Napsin A	/	/	++
TTF-1	++	++++	++
Ki-67	20%	40%	15%
CK7	/	++++	/
P40	−	−	−
Syn	−	+	−
CR	−	−	−
WT-1	/	/	−

+: positive, −: negative, /: the test has not been carried out.

Upon admission, a closed drainage procedure for the thoracic cavity was urgently performed to treat the left-sided pleural effusion, resulting in improved hypoxia symptoms and overall condition. The patient’s Performance Status (PS) score decreased from 2 at admission to 1. Based on a comprehensive review of published case reports concerning this rare EGFR L747P mutation and taking into consideration the patient’s financial constraints alongside local medical insurance policies, gefitinib was selected as the first-line treatment regimen, gefitinib therapy was prescribed for the patient after confirmation of LUAD harboring EGFR L747P mutation on November 4, 2022 (Figures 1A, B). An unadjusted anti-rejection regimen was followed before discharge. The patient remained stable on radiography for 9 months (Figures 1C–F) until follow-up chest enhanced CT on August 5, 2023 indicated progression (Figure 1G). During the gefitinib treatment follow-up, tumor markers CEA and Cyfra 21-1 levels increased over time (Figure 2C). From September 16, 2023 onward, osimertinib (80 mg/d) was administered as a second-line drug due to disease progression (Figure 1H). At the November evaluation (Figure 1I), the target lesion diameter (sum of the longest diameters, SLD) had decreased with no new lesions observed, indicating stable disease (SD). By January 2014, however, both an increase in the target lesion diameter (SLD) and the emergence of new nodules were noted, consistent with progressive disease (PD) (Figure 1J). On February 2, 2024, dacomitinib (30 mg/d) replaced osimertinib as third-line treatment resulting in significant shrinkage of metastatic tumors in the left pleura and right lung found by chest CT on March 3, 2024 (Figure 1K). However, follow-up of a chest CT on April 25, 2024, showed that although the target lesion diameter (SLD) had decreased slightly from 51 to 50 mm, new lesions appeared, consistent with progressive disease (PD). This finding suggests mixed response to dacomitinib (Figure 1L). Urea nitrogen and creatinine remained within normal range during follow-up until March 2024; however, hematuria with a grade of +2 was initially detected on December10, 2022 progressing to +3 on August 4, 2023 with microscopy identifying glomerular hematuria while urinary proteinuria first appeared on March 4, 2024. Hematuria and proteinuria suggest endothelial injury caused by TKIs, the transplant specialist adjusted the immune transplantation regimen of patients from December 2023 to tacrolimus (0.5 mg, twice daily), prednisolone (5 mg, once daily), and sirolimus (0.5 mg and 1 mg, alternating once a day). The patient tolerated TKIs well, complicated by manageable grade 1 paronychia when using gefitinib and dacomitinib. The patient was lost to follow-up in June 2024 and subsequently passed away in November 2024, achieving an OS of 24 months. Throughout EGFR-TKI therapy, the patient maintained a strict vegetarian diet (excluding all animal protein due to Buddhist beliefs), relying on plant-based sources like soy and nuts. Physical activity was limited to light walking, consistent with his stable PS of 1 after initial improvement. No specific environmental modifications were documented. The patient was still receiving a third-generation TKI during the period of loss to follow-up, but the specific medication was unknown; the ultimate cause of death was also unclear due to the loss to follow-up.

## Discussion

Lung cancer incidence is markedly higher in organ transplant recipients than in the general population (14, 15). Adenocarcinoma is the most common subtypes, consistent with patterns observed in non-transplant patients (16). In our case, risk factors included female sex, age over 35 years at transplantation, and long-term use of calcineurin inhibitors (>16 years). The patient presented with breathing difficulties due to malignant pleural effusion and was diagnosed with stage IV LUAD. As lung cancer is a leading cause of cancer-related death in KTRs, but early-stage disease remains highly curable, enhanced vigilance and early screening are warranted. Unfortunately, our patient was diagnosed at an advanced stage, missing the opportunity for early intervention. Pulmonary nodules detected on chest CT in this population should be carefully differentiated from infectious diseases such as tuberculosis or fungal infections, and the possibility of concurrent infection and malignancy must also be considered.

The main challenge in this case was managing advanced LUAD in a renal transplant recipient on long-term immunosuppressive therapy. For advanced NSCLC, first-line treatment typically involves TKIs targeting driver alterations such as EGFR mutations or ALK/ROS1 rearrangements (17). However, the EGFR L747P mutation is rare, and no consensus guidelines exist. Treatment decisions were therefore guided by published case reports. Preclinical studies suggest that the L747P mutation enhances tumorigenic potential and may confer resistance to TKIs (10). Some reports describe intrinsic resistance to first-generation [erlotinib (18), gefitinib (6)] and third-generation [osimertinib (6)] TKIs, whereas others have documented responses to agents across all three generations (7–12). Computational modeling indicates that second-generation TKIs may have stronger binding affinity to L747P than first- or third-generation agents (16), but clinical evidence remains inconsistent.

According to a previous case report, gefitinib achieved a PFS of 18 months, followed by a short period of clinical benefit from osimertinib (9). Drawing on this evidence, and considering both therapeutic efficacy and economic feasibility within the reimbursement policy, our patient adopted a similar treatment strategy. Gefitinib was initiated as first-line therapy and achieved a PFS of 8 months, which was shorter than previously reported. Upon disease progression, second-line osimertinib provided disease control for 4.5 months, consistent with outcomes described in the literature. Subsequently, dacomitinib was administered as third-line therapy, inducing an initial radiographic response; however, rapid progression occurred after 2.5 months, suggesting early resistance. The resistance mechanism could not be clarified, as no repeat biopsy was performed. The patient was later lost to follow-up, and according to family reports, she continued treatment with an unspecified third-generation TKI at another institution before succumbing to disease approximately 6 months thereafter.

During first-line gefitinib treatment, we observed biochemical progression with rising CYFRA21-1 and CEA levels despite radiographic stability. CYFRA21-1 is a recognized NSCLC biomarker, but it is more sensitive in squamous cell carcinoma than in adenocarcinoma. The unexpected increase in both markers raises important questions about tumor biology. Possible explanations include: (i) tumor heterogeneity or mixed histology (e.g., adenosquamous carcinoma) not captured by the initial biopsy; (ii) clonal evolution or phenotypic switch under TKI treatment pressure; and (iii) altered biomarker kinetics influenced by the immunocompromised state of the transplant recipient. While adenocarcinoma remained the confirmed histopathological diagnosis, this discordance highlights the complexity of tumor biology and the potential value of biomarker dynamics in monitoring therapeutic response.

Managing cancer in KTRs is particularly challenging because of the need to preserve allograft function while accounting for interactions with immunosuppressive therapy (19). TKIs have been associated with hypertension and proteinuria, likely due to reduced nitric oxide production and endothelial injury (20). Therefore, routine monitoring of blood pressure and urinalysis is essential, as demonstrated in our case. Since immunosuppression contributes to the development of post-transplant malignancies, adjustment or reduction of immunosuppressive agents remains a cornerstone of cancer management in this population. In general, tapering or discontinuation of glucocorticoids is recommended, with maintenance on calcineurin inhibitors or mammalian target of rapamycin inhibitor (mTORi) (21). At the time of LUAD diagnosis, the patient was receiving prednisone (5 mg daily), tacrolimus (0.5 mg twice daily), and mycophenolate mofetil (250 mg twice daily). This low-dose immunosuppressive regimen showed no significant drug interactions with EGFR-TKIs, so it was continued after initiation of gefitinib. During follow-up, urinalysis revealed hematuria at 2 months and progression at 7 months, likely related to endothelial injury from gefitinib, although renal function remained stable. Consequently, no adjustment was made during gefitinib or osimertinib treatment. Upon switching to dacomitinib, the dose was reduced to two-thirds of the standard (30 mg) for renal protection. Proteinuria was first detected 1 month later, again suggesting TKI-related renal toxicity. At the time of manuscript submission, serum creatinine remained within the normal range.

Proteinuria and hematuria may also result from rejection or recurrence of the primary kidney disease. However, in this case, their temporal onset following TKI initiation strongly supports a drug-related cause. Throughout follow-up, renal function (serum creatinine) remained stable, immunosuppressant levels were within the therapeutic range, and no clinical signs of acute rejection (e.g., fever, graft tenderness) or urinary tract infection were observed. Considering these factors and the well-documented endothelial and renal toxicities of TKIs, we conclude that TKI-induced injury was the most likely etiology.

## Conclusion

This case highlights the importance of vigilant cancer surveillance in KTRs, who are at increased risk of malignancies. EGFR L747P mutations show inconsistent and short-lived responses to different TKIs, and treatment decisions in transplant recipients are particularly challenging due to the need to balance cancer control with immunosuppression. Our case demonstrates that sequential use of multiple TKIs can provide meaningful survival benefit, underscoring the need for individualized therapeutic strategies. Further clinical evidence is required to establish standardized treatment recommendations for this rare mutation in transplant recipients.

## Data Availability

The original contributions presented in this study are included in this article/supplementary material, further inquiries can be directed to the corresponding authors.
